# Mood Profiling in Singapore: Cross-Cultural Validation and Potential Applications of Mood Profile Clusters

**DOI:** 10.3389/fpsyg.2020.00665

**Published:** 2020-04-09

**Authors:** Christie S. Y. Han, Renée L. Parsons-Smith, Peter C. Terry

**Affiliations:** ^1^School of Psychology and Counselling, University of Southern Queensland, Toowoomba, QLD, Australia; ^2^School of Social Sciences, University of the Sunshine Coast, Sippy Downs, QLD, Australia; ^3^Division of Research and Innovation, University of Southern Queensland, Toowoomba, QLD, Australia

**Keywords:** affect, emotion, cluster analysis, mood profiling, BRUMS

## Abstract

Mood profiling is a popular method of quantifying and classifying feeling states. Previous research has identified several novel mood profiles in predominantly Western English-speaking populations (Parsons-Smith et al., 2017), and replicated the findings in the domain of sport and exercise (Quartiroli et al., 2018; Terry and Parsons-Smith, 2019). The aim of the current study was to investigate if six hypothesized clusters of mood responses were evident in a population of English-speaking sport and non-sport participants in Singapore. A seeded k-means cluster analysis was applied to the mood responses of 1,444 participants (991 male, 440 female, 13 unspecified; aged 18–65 years) who completed the Brunel Mood Scale (BRUMS; Terry et al., 1999, 2003a). The six hypothesized mood profiles (i.e., iceberg, inverse Everest, inverse iceberg, shark fin, submerged, and surface profiles) were identified clearly. Chi-squared analyses showed unequal distribution of the profiles by gender, age group, ethnicity, education level, and sport participation. Findings support the cross-cultural generalizability of the six mood profiles in English-speaking sport and non-sport samples in Singapore and contribute to investigation into the antecedents, correlates, and consequences of each mood profile.

## Introduction

Sport psychologists have long been interested in the study of mood and its relationship with performance. For the purpose of our investigation, mood is defined as “a set of feelings, ephemeral in nature, varying in intensity and duration, and usually involving more than one emotion” (Lane and Terry, 2000, p. 17). Morgan (1980) reported that mood responses were predictive of athletic performance and developed a mental health model proposing that positive mood is associated with psychological well-being and athletic success, whereas negative mood is associated with psychopathology and poor performance (Morgan, 1985). The notion that the *iceberg* profile, a pattern of mood responses characterized by above average vigor and below average tension, depression, anger, fatigue, and confusion, was the “test of champions” (Morgan, 1980) gained much traction and spawned a large body of research on the subject (see Rowley et al., 1995; Beedie et al., 2000). Research subsequently showed that the iceberg profile was the typical mood profile reported by athletes, successful or otherwise, and hence its predictive effectiveness may be more limited than previously claimed (Renger, 1993; Terry and Lane, 2000).

Athletic performance for some individuals is closely related to mood but this is not the case for other individuals (Totterdell, 1999; Lane and Chappell, 2001) and therefore the utility of mood profiling for predicting performance may rely on the individualized assessment of idiosyncratic mood-performance relationships (Terry, 1995). Other applications of mood profiling in sport include assessing risk of burnout by overtraining (Morgan et al., 1987), screening for risk of eating disorders (Terry and Galambos, 2004), quantifying the beneficial effects of music (Terry et al., 2020), and as a catalyst for discussion between practitioner and athlete to gain insights into performance and well-being (Terry, 1995).

In addition to the iceberg profile, other distinct mood profiles have been identified. The *inverse iceberg* profile, characterized by below average scores for vigor and above average scores for tension, depression, anger, fatigue, and confusion, has been shown to be associated with reduced performance (Budgett, 1998), higher occurrence of athletic injury (Galambos et al., 2005), and risk of mental health disorders (van Wijk et al., 2013). The *Everest* profile, a more prominent iceberg profile characterized by higher vigor scores, and lower tension, depression, anger, fatigue, and confusion scores, is anecdotally associated with superior performance (Terry, 1995).

More recent studies (Parsons-Smith et al., 2017; Quartiroli et al., 2018) have identified new profiles, referred to as the *inverse Everest*, *surface*, *shark fin*, and *submerged* profiles. As described by Parsons-Smith et al. (2017, pp. 4–5), the inverse Everest profile is characterized by low vigor scores, high scores for tension and fatigue, and very high scores for depression, anger, and confusion. The surface profile is characterized by average scores for all six mood dimensions. The shark fin profile is characterized by below average scores for tension, depression, anger, vigor, and confusion, combined with a high score for fatigue. The submerged profile is characterized by below average scores for all six mood dimensions.

To date, research on mood profile clusters has been limited largely to Western populations. In order to demonstrate the cross-cultural stability and generalizability of clusters, it is necessary to identify them in different cultures. Singapore is a multicultural society whose citizens are mainly of Asian descent, with the main ethnic groups being Chinese, Malay, and Indian. Singapore’s multiculturalism is reflected in its official documents and public signs, which are available in English, Mandarin, Malay, and Tamil. English is the official language of the country, largely due to acculturation from previous British colonial rule; hence English serves as the official medium of instruction in schools and businesses (Tanzer et al., 1996). As such, the large majority of Singaporeans, especially those educated in the public schools since the 1980s, are fluent in the English language.

Our study had two aims: (1) To examine if mood profile clusters previously reported are also evident in the general Singapore English-speaking population; and (2) to examine the distribution of distinct mood profiles across various demographic variables of interest. Retrieving the hypothesized mood profile clusters in a Singaporean sample will provide further support for the validity and generalizability of these mood profiles, demonstrating their cross-cultural replicability. Mood profiling as an applied technique has considerable potential for practitioners to examine relationships between mood and various outcomes of interest in clinical as well as non-clinical settings. Clarification of the distribution of mood profiles in relation to various demographic variables may provide insights into the design and implementation of potential interventions and programs to better serve various segments of the Singaporean population. Results from this study set the stage for further research in Singapore to explore the practical, clinical, and theoretical implications of mood profiling.

## Materials and Methods

### Participants

A total of 1,444 Singapore residents participated in the study (991 male, 440 female, 13 unspecified; age range: 18–65 years). The ethnic distribution of participants was 80.5% Chinese, 8.2% Malay, 8.2% Indian, and 2.1% other, which approximates the ethnic distribution of Singapore as a whole (Department of Statistics Singapore, 2018). All participants could read and write in English, which is the first language of most Singaporeans. A total of 65.4% (*n* = 945) of respondents participated in sport either for leisure (*n* = 514) or competitively (*n* = 431). Table 1 presents the sample demographics.

**TABLE 1:** Sample demographics (*N* = 1,444).

**Source**	***n***	**%**
**Gender**		
Male	991	68.6
Female	440	30.5
Unspecified	13	<1
**Age band**		
18–21	507	35.1
22–25	256	17.7
26–30	336	23.3
31–35	140	9.7
36–40	75	5.2
41–45	34	2.4
46–50	22	1.5
51–55	24	1.7
>55	36	2.5
Unspecified	14	1.0
**Age group**		
≤25 years	763	52.8
26+ years	667	46.2
Unspecified	14	1.0
**Ethnicity**		
Chinese	1163	80.5
Malay	119	8.2
Indian	118	8.2
Other	31	2.1
Unspecified	13	<1
**Education level**		
Primary/Secondary	70	4.8
JC/IB/Poly/ITE^#^	873	60.5
University	370	25.6
Postgraduate	93	6.4
Other	18	1.2
Unspecified	20	1.4
**Sport participation**		
Sport	954	66.1
Non-sport	476	33.0
Unspecified	14	1.0
**Participation level**		
Recreational	514	35.6
School/Club	339	23.5
Regional/International	92	6.4
Unspecified	9	<1

*^#^JC, Junior College; IB, International Baccalaureate; Poly, Polytechnic Diploma; ITE, Institute of Technical Education Certification.*

### Measures

Participants reported relevant demographic information (gender, age band, ethnicity, education level, sport participation, participation level) and completed a mood scale. Mood was assessed using the Brunel Mood Scale (BRUMS; Terry et al., 1999, 2003a), a 24-item scale of basic mood descriptors, in its original format with the recommended response timeframe of “How do you feel right now?” (Terry et al., 2005). Participants rated their responses on a five-point Likert scale (0 = *not at all*, 1 = *a little*, 2 = *moderately*, 3 = *quite a bit*, and 4 = *extremely*). The measure has six subscales (i.e., anger, confusion, depression, fatigue, tension, and vigor) each with four items, with total subscale scores ranging from 0 to 16. No total mood disturbance scores were calculated, as the focus of the study was on the profiles generated from the six subscale scores. The BRUMS has been validated across diverse cultures (e.g., Terry et al., 2003b; Zhang et al., 2014) and contexts (e.g., van Wijk et al., 2013; Sties et al., 2014). Subscales show good internal consistency, with Cronbach alpha coefficients ranging from 0.74 to 0.90 (Terry et al., 1999). Cross-cultural assessment of the BRUMS has supported it as a valid measure of mood in the Singaporean context, and local norms are available (Han et al., 2019).

### Procedure

Two methods of data collection were used to recruit English-speaking Singaporean participants. First, a link to an online version of the BRUMS, hosted on the university’s eResearch Survey System was distributed to a sample of Singapore residents using snowball sampling and assistance from the Singapore Sport Institute, which produced a sample of 695 respondents (48.1% of the total sample). A second group of participants was recruited at random from a range of organizations (e.g., schools and workplaces) who completed a paper-and-pencil version of the BRUMS. Participants were informed they were part of a research study aimed at validating a mood scale for use in Singapore, and their informed consent was sought prior to participation in the study. This study was conducted in accordance with the Australian Code for the Responsible Conduct of Research. The protocol was approved by the Human Research Ethics Committee at the University of Southern Queensland (USQ; approval number: H17REA143).

### Data Screening

A total of 13 cases with missing values for the BRUMS were excluded from the analysis. Data were screened for compliance with the assumptions of univariate and multivariate normality. Like previous samples (Parsons-Smith et al., 2017), significant univariate non-normality was found in some subscales (e.g., depression, anger, and tension), consistent with typical mood subscale distributions. Negative mood scores tended toward higher numbers at the lower end, and lower numbers at the upper end (Terry et al., 1999). The frequency distributions for skewness and kurtosis were examined and it was concluded that deviations from normal distribution were unlikely to make a substantive difference to the analyses, thus no data were removed. A total of 45 multivariate outliers were identified according to a Mahalanobis distance statistic at *p* < 0.001. A case-by-case visual inspection suggested that response patterns were plausible. Given this, all outliers were retained, leaving a final sample of 1,444 respondents.

## Results

The results of this study are presented in three parts–cluster analysis, discriminant function analysis, and distribution of mood profiles by demographic variables.

### Cluster Analysis

**FIGURE 1:**
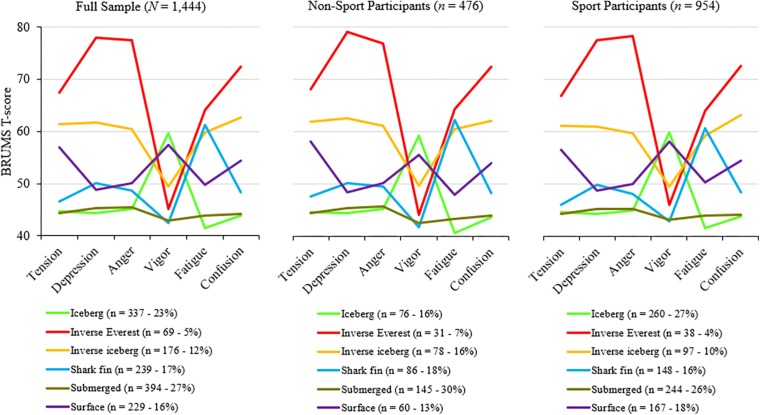
Visual representations of the six mood profiles.

The objective of cluster analysis was described by Anderberg (2014) as the separation of data units or variables into groups (i.e., clusters), such that the elements within a cluster have a high degree of natural association and the clusters are relatively distinct from one another. The Statistical Package for Social Sciences, version 25 was used to conduct the cluster analysis, specifically *k*-means clustering (MacQueen, 1967), a method used to automatically split a dataset into a specified number of clusters. Using raw score cluster centroids from Quartiroli et al. (2018) (see Table 2), a seeded *k*-means cluster analysis with a prescribed six-cluster solution was run on the whole sample, and then separated by sport and non-sport participants. The six clusters previously identified by Parsons-Smith et al. (2017) and Quartiroli et al. (2018), referred to as the iceberg profile (23.3% of the sample), inverse Everest profile (4.8%), inverse iceberg profile (12.2%), shark fin profile (16.6%), submerged profile (27.3%), and surface profile (15.9%), were clearly identified in the present sample. Descriptive statistics of the six-cluster solution are presented in Table 3, presented as *T*-scores derived from local norms provided by Han et al. (2019). The distribution of clusters is similar to that reported by Parsons-Smith et al. (2017). Visual representations of the six mood profiles are shown in Figure 1.

**TABLE 2:** Raw score centroids used in the cluster analysis.

	Cluster					
Mood dimension	1	2	3	4	5	6
Tension	1.15	10.42	6.34	1.75	1.29	4.58
Depression	0.25	11.19	5.11	1.26	0.59	1.69
Anger	0.41	10.23	4.52	0.95	0.48	2.26
Vigor	10.62	4.69	5.98	4.14	4.72	9.10
Fatigue	2.39	11.83	8.59	9.97	2.91	4.76
Confusion	0.54	10.75	5.84	1.32	0.90	3.27

*1 = iceberg, 2 = inverse Everest, 3 = inverse iceberg, 4 = shark fin, 5 = submerged, 6 = surface.*

**TABLE 3:** Descriptive statistics of the 6-cluster solution (*N* = 1,444).

	**Iceberg**			**Inverse Everest**		
	**(*n* = 337, 23.3%)**			**(*n* = 69, 4.8%)**		
**Mood dimension**	***M***	***SD***	**95% CI**	***M***	***SD***	**95% CI**

Tension	44.65	4.37	[44.18, 45.12]	67.40	12.35	[64.44, 70.37]
Depression	44.30	2.87	[44.00, 44.61]	78.06	7.97	[76.15, 79.98]
Anger	45.08	3.42	[44.72, 45.45]	77.52	11.55	[74.75, 80.30]
Vigor	59.70	6.04	[59.05, 60.35]	45.20	9.49	[42.92, 47.48]
Fatigue	41.43	4.97	[40.89, 41.96]	64.10	6.97	[62.43, 65.78]
Confusion	43.83	4.13	[43.39, 44.27]	72.42	8.76	[70.31, 74.52]

	**Inverse iceberg**			**Shark fin**		
	**(*n* = 176, 12.2%)**			**(*n* = 239, 16.6%)**		
**Mood dimension**	***M***	***SD***	**95% CI**	***M***	***SD***	**95% CI**

Tension	61.40	9.20	[60.03, 62.77]	46.64	5.90	[45.89, 47.39]
Depression	61.69	8.17	[60.47, 62.90]	50.06	7.30	[49.13, 50.99]
Anger	60.38	9.58	[58.95, 61.80]	48.65	6.41	[47.84, 49.47]
Vigor	49.52	8.14	[48.31, 50.73]	42.48	6.94	[41.59, 43.36]
Fatigue	59.78	7.09	[58.72, 60.83]	61.27	5.28	[60.60, 61.94]
Confusion	62.68	8.22	[61.46, 63.90]	48.35	6.41	[47.53, 49.17]

	**Submerged**			**Surface**		
	**(*n* = 394, 27.3%)**			**(*n* = 229, 15.9%)**		
**Mood dimension**	***M***	***SD***	**95% CI**	***M***	***SD***	**95% CI**
Tension	44.43	4.65	[43.97, 44.89]	56.96	8.50	[55.85, 58.07]
Depression	45.38	4.16	[44.96, 45.79]	48.84	5.92	[48.07, 49.61]
Anger	45.54	4.08	[45.13, 45.94]	50.05	7.03	[49.14, 50.97]
Vigor	42.99	5.72	[42.42, 43.55]	57.46	6.94	[56.56, 58.36]
Fatigue	43.80	4.83	[43.32, 44.27]	49.77	5.63	[49.03, 50.50]
Confusion	44.17	4.54	[43.72, 44.62]	54.33	6.45	[53.49, 55.17]

*Table presents *T*-scores converted from raw scores with reference to local norms reported by Han et al., 2019.*

### Discriminant Function Analysis

To explore the clusters further, a *post hoc* simultaneous multiple discriminant function analysis (DFA) was conducted to evaluate how well the clusters were classified. DFA predicts group membership in naturally occurring groups, based on predictor variables, and is not affected by unequal sample sizes (Tabachnick and Fidell, 2013). DFA is a two-step statistical procedure which involves significance testing of discriminant functions, followed by a computational process of classification. The ratio of cases to independent variables was 241 to 1, which far exceeds the recommended minimum ratio of 20 to 1. The DFA identified five functions accounting for 100% of the variance with the first three functions accounting for 98.2% of the variance (Table 4). A single-factor MANOVA was conducted to assess the between-cluster differences in subscale scores, which showed a significant omnibus effect, Wilks’ λ = 0.031, *F*(30,5734) = 266.04 *p* < 0.001, partial η^2^ = 0.502. Using a Bonferroni-adjusted alpha level of 0.008, significant univariate effects were confirmed for all mood dimensions: tension, *F*(5,1438) = 352.14, *p* < 0.001, partial η^2^ = 0.550; depression, *F*(5,1438) = 609.78, *p* < 0.001, partial η^2^ = 0.680; anger, *F*(5,1438) = 444.98, *p* < 0.001, partial η^2^ = 0.607; vigor, *F*(5,1438) = 347.85, *p* < 0.001, partial η^2^ = 0.547; fatigue, *F*(5,1438) = 667.34, *p* < 0.001, partial η^2^ = 0.699; confusion, *F*(5,1438) = 539.79, *p* < 0.001, partial η^2^ = 0.652.

**TABLE 4:** Discriminant functions (*N* = 1,444).

Discriminant function	Eigenvalue	% of variance	Cumulative%	Canonical correlation
1	6.373	74.2	74.2	0.930
2	1.551	18.1	92.3	0.780
3	0.507	5.9	98.2	0.580
4	0.151	1.8	100.0	0.363
5	0.003	0.0	100.0	0.055

Mood dimensions strongly associated with Function 1 were high depression, fatigue, confusion, anger, and tension. Function 2 identified high vigor and tension, and low fatigue. Function 3 identified low depression and anger, and high fatigue and vigor. Table 5 lists all five discriminant functions. The findings suggest that the distribution overlap was small and the functions discriminated between clusters with a very high degree of accuracy, with correct classifications for the iceberg profile at 99.1%, inverse iceberg profile at 89.9%, inverse iceberg at 94.3%, shark fin profile at 95.4%, submerged profile at 99.5%, and surface profile at 91.7% (see Table 6). It was found that 96.4% of the cases were correctly reclassified back into the original clusters, which is markedly higher than the minimum classification accuracy rate of 44.9% (i.e., the proportional by chance accuracy +25%).

**TABLE 5:** Structure matrix (*N* = 1,444).

	Discriminant function				
Mood dimension	1	2	3	4	5
Tension	0.388	0.349	–0.030	−0.701*	0.079
Depression	0.558*	0.091	–0.411	0.509	–0.445
Anger	0.469	0.165	–0.407	0.371	0.662*
Vigor	–0.104	0.808*	0.473	0.302	0.137
Fatigue	0.529	–0.417	0.720*	0.139	0.088
Confusion	0.520*	0.282	–0.117	–0.376	–0.311

*Largest absolute correlation between each variable and any discriminant function.

**TABLE 6:** Cluster classifications (*N* = 1,444).

	Cluster							
Cluster	1	2	3	4	5	6	*n*	%
Iceberg	334	0	0	0	3	0	337	99.1
Inverse Everest	0	62	7	0	0	0	69	89.9
Inverse iceberg	0	0	166	8	0	2	176	94.3
Shark fin	0	0	5	228	6	0	239	95.4
Submerged	1	0	0	0	392	1	394	99.5
Surface	11	0	2	0	6	210	229	91.7

*1 = iceberg, 2 = inverse Everest, 3 = inverse iceberg, 4 = shark fin, 5 = submerged, 6 = surface.*

### Demographic Influences on Mood Responses

Single-factor MANOVAs were used to provide an initial assessment of the influence of demographic variables on mood responses. Participants in the unspecified and “other” categories were excluded from these analyses. Significant multivariate variability was found for gender, age group, ethnicity, education level, and sport participation (see Table 7) but not for participation level, which was excluded from subsequent analyses. Univariate analyses identified many significant between-group differences. Females reported higher scores for anger, confusion, depression, and fatigue, and lower scores for vigor, compared to males. Younger participants reported higher scores for confusion and tension than older participants. Malay participants reported higher anger, confusion, depression, fatigue, and tension scores than Chinese participants. Participants with up to secondary education reported lower scores for fatigue and higher scores for vigor than those with a university education. Those involved in sport reported lower scores for anger, depression, fatigue, and tension, and higher scores for vigor, than those not involved in sport.

**TABLE 7:** MANOVA of BRUMS subscales by demographic variables.

	Tension		Depression		Anger		Vigor		Fatigue		Confusion	
Source	*M*	*SD*	*M*	*SD*	*M*	*SD*	*M*	*SD*	*M*	*SD*	*M*	*SD*
**Gender *F*(6, 1424) = 17.52^†^**												
Male (*n* = 991)	49.63	9.74	48.57^†^	8.89	48.84^†^	8.76	50.99^†^	10.00	48.69^†^	9.45	49.27^†^	9.25
Female (*n* = 440)	50.89	10.60	53.19	11.57	52.64	12.00	47.96	9.68	52.90	10.59	51.67	11.39
**Age group *F*(6, 1423) = 11.54^†^**												
≤25 years (*n* = 763)	51.35^†^	10.82	49.96	9.75	49.63	9.44	49.78	10.15	50.61	9.95	50.91^†^	10.21
26+ years (*n* = 667)	48.50	8.79	50.04	10.33	50.43	10.64	50.35	9.80	49.27	10.03	48.99	9.70
**Ethnicity *F*(12, 2784) = 6.43^†^**												
Chinese^a^ (*n* = 1,163)	49.57	9.73	49.42	9.45	49.43	9.44	49.57*^c^	9.82	49.88	9.75	49.68	9.72
Malay^b^ (*n* = 119)	53.89^†a^	11.35	54.81^†a^	12.35	55.00^†a^	12.70	51.15	10.58	53.95^†*ac*^	11.19	53.63^†a^	11.82
Indian^c^ (*n* = 118)	51.56	11.43	51.46	11.77	51.17*^b^	11.36	53.03	10.58	47.94	10.34	50.34	10.74
**Level of education *F*(18, 3951) = 4.52^†^**												
Primary/Secondary^a^ (*n* = 70)	50.29	9.88	50.04	9.92	50.03	9.17	54.75*^b^	10.56	45.92*^bc^	9.08	48.71	8.87
JC/IB/Poly/ITE^#b^ (*n* = 873)	50.82	10.44	49.56	9.61	49.60	9.73	50.12	9.97	50.20	10.05	50.44	10.01
University^c^ (*n* = 370)	48.86	9.35	51.12	10.81	50.76	10.76	48.67^†a^	9.63	50.71	10.23	49.61	10.39
Postgraduate^d^ (*n* = 93)	48.09	8.49	49.35	10.11	50.35	10.31	50.88	10.12	48.72	9.13	49.09	9.47
**Sport participation *F*(6, 1423) = 8.86^†^**												
Sport (*n* = 954)	49.41*	9.48	49.20^†^	9.33	49.27^†^	9.31	51.07^†^	10.07	49.43*	9.58	49.61	9.73
Non-sport (*n* = 476)	51.22	10.96	51.52	11.08	51.46	11.20	47.99	9.52	51.11	10.74	50.81	10.54

Table presents *T*-scores converted from raw scores with reference to local norms reported by Han et al., 2019. ^#^JC = Junior College, IB = International Baccalaureate, Poly = Polytechnic Diploma, ITE = Institute of Technical Education Certification. *p < 0.008, ^†^*p* < 0.001.

### Distribution of Mood Profiles by Demographic Variable

Chi-squared tests were used to assess the distribution of mood profile clusters according to the demographic variables of interest. Significant variations in distribution were evident (see Table 8), for gender, age group, ethnicity, level of education, and sport participation. Adjusted residuals were used to investigate the source of these differences. The distribution of mood profiles by the various demographic variables are elaborated below.

**TABLE 8:** Distribution of clusters by demographic variables.

	Cluster											
**Source**	**1**	**%**	**2**	**%**	**3**	**%**	**4**	**%**	**5**	**%**	**6**	**%**
**Gender χ^2^(5, 1431) = 80.81^†^**												
Male (*n* = 991)	256^§+^	25.8	31^†–^	3.1	94^†–^	9.5	137^†–^	13.8	285*^+^	28.8	188^†+^	19.0
Female (*n* = 440)	80^§–^	18.2	38^†+^	8.6	81^†+^	18.4	97^†+^	22.0	104*^–^	23.6	40^†–^	9.1
**Age group χ^2^(5, 1430) = 25.78^†^**												
≤25 years (*n* = 763)	148^†–^	19.4	32	4.2	110^§+^	14.4	127	16.6	205	26.9	141^§+^	18.5
26+ years (*n* = 667)	187^†+^	28.0	37	5.5	65^§–^	9.7	107	16.0	184	27.6	87^§–^	13.0
**Ethnicity χ^2^(10, 1400) = 38.49^†^**												
Chinese (*n* = 1,163)	266	22.9	46^§–^	4.0	131^§–^	11.3	200	17.2	335^§+^	28.8	185	15.9
Malay (*n* = 119)	24	20.2	14^§+^	11.8	25^§+^	21.0	18	15.1	18^§–^	15.1	20	16.8
Indian (*n* = 118)	36	30.5	7	5.9	18	15.3	12	10.2	26	22.0	19	16.1
**Level of education χ^2^(15, 1406) = 39.77^†^**												
Primary/Secondary (*n* = 70)	23*^+^	32.9	3	4.3	4	5.7	5*^–^	7.1	16	22.9	19^§+^	27.1
JC/IB/Poly/ITE^#^ (*n* = 873)	189	21.6	37	4.2	112	12.8	139	15.9	237	27.1	159^§+^	18.2
University (*n* = 370)	87	23.5	24	6.5	46	12.4	76*^+^	20.5	102	27.6	35^†–^	9.5
Postgraduate (*n* = 93)	28	30.1	5	5.4	9	9.7	11	11.8	28	30.1	12	12.9
**Sport participation χ^2^(5, 1430) = 40.32^†^**												
Sport (*n* = 954)	260^†+^	27.3	38*^–^	4.0	97^†–^	10.2	148	15.5	244*^–^	25.6	167*^+^	17.5
Non-sport (*n* = 476)	76^†–^	16.0	31*^+^	6.5	78^†+^	16.4	86	18.1	145*^+^	30.5	60*^–^	12.6

1 = iceberg, 2 = inverse Everest, 3 = inverse iceberg, 4 = shark fin, 5 = submerged, 6 = surface. ^#^JC = Junior College, IB = International Baccalaureate, Poly = Polytechnic Diploma, ITE = Institute of Technical Education Certification. *p < 0.05, ^§^*p* < 0.01, ^†^*p* < 0.001. ^+^over-represented, ^–^under-represented.

#### Gender

The distribution of the six mood profiles varied significantly by gender for all profiles. Males were over-represented in the iceberg, submerged, and surface profiles, whereas females were over-represented in the inverse Everest, inverse iceberg, and shark fin profiles, consistent with the findings of Parsons-Smith et al. (2017) and Quartiroli et al. (2018).

#### Age Group

The distribution of inverse Everest, shark fin, and submerged profiles was independent of age group. Younger participants (≤25 years) were over-represented in the inverse iceberg and surface profiles and under-represented for the iceberg profile. Older participants (26+ years) were over-represented in the iceberg profile and under-represented in the inverse iceberg and surface profiles.

#### Ethnicity

The distribution of iceberg, shark fin, and surface profiles was independent of ethnicity. Chinese Singaporeans were over-represented in the submerged profile and under-represented in the inverse Everest and inverse iceberg profiles. Malay Singaporeans were over-represented in inverse Everest and inverse iceberg profiles and under-represented in the submerged profile.

#### Level of Education

The distribution of the inverse Everest, inverse iceberg, and submerged profiles was independent of level of education. Participants with secondary school or below level of education were over-represented in the iceberg and surface profiles and under-represented in the shark fin profile. Participants with pre-university education (JC/IB/Poly/ITE) were over-represented in the surface profile, whereas those with a university education were over-represented in the shark fin profile and under-represented in the surface profile.

#### Sport Participation

The distribution for the shark fin profile was independent of sport participation. Those who participated in sport were over-represented in the iceberg and surface profiles and under-represented in the inverse Everest, inverse iceberg, and submerged profiles. Those who did not participate in sport were over-represented in the inverse Everest, inverse iceberg, and submerged profiles and under-represented in the iceberg and surface profiles.

## Discussion

Six mood profile clusters previously identified by Parsons-Smith et al. (2017) and Quartiroli et al. (2018), referred to as the iceberg, inverse iceberg, inverse Everest, shark fin, submerged, and surface profiles, were clearly retrieved among our sample of Singaporean participants, showing a similar structure matrix.

Support for the cross-cultural generalizability of these mood profiles makes a significant contribution to the literature on mood profiling and, more specifically, to the validation of the BRUMS as a tool for mood profiling in Singapore. The identification of associations between demographic variables and the incidence of mood profiles across multiple studies provides support for the use of mood profiling for various purposes, ranging from screening or monitoring of mental health to providing a tool to predict performance or other outcome variables of interest. Our findings enhance understanding of the association between demographic variables and mood profiles and may serve as a guide for practitioners and policy makers in the design of potential interventions to promote well-being among general as well as clinical populations.

A noteworthy finding of our study was that men were over-represented in the more positive iceberg profile whereas females were over-represented in the more negative inverse Everest, inverse iceberg, and shark fin profiles. There are several possible explanations for this gender difference. First, it has been reported that men and women regulate mood differently. For example, women are more likely to ruminate that men, which may influence their reported mood (Nolen-Hoeksema and Jackson, 2001). Second, there is a gender difference in hormonal activity (e.g., premenstrual syndrome, postnatal depression, menopause; Halbreich and Kahn, 2001) which may make some women more prone to negative moods. Third, it has been reported that women are more likely than men to report feeling depressed, sad, anxious, or nervous (American Psychological Association, 2012b), potentially explaining why women were over-represented in the more negative mood profiles. Finally, males are often taught from a young age to inhibit expression of emotion to a greater extent than females (Underwood et al., 1992), which may explain why males were over-represented for the submerged profile which has low scores on all six dimensions of mood.

In relation to age group, younger participants were under-represented while older participants were over-represented for the iceberg profile, whereas the reverse was true for the inverse iceberg profile. This is similar to trends identified in previous investigations of the age-related incidence of mood profile clusters (Parsons-Smith et al., 2017; Quartiroli et al., 2018). Interestingly, the age of onset of mood disorders is typically around the mid-20s (Kessler et al., 2007) and data from the *Stress in America* survey showed younger people report higher stress levels than older generations and also report that they do not manage it well (American Psychological Association, 2012a). These age-related trends in mood responses are worthy of further investigation in a Singaporean context.

With regard to ethnicity, our findings showed a higher incidence of negative mood profiles (inverse Everest, inverse iceberg) among Malay Singaporeans and a lower incidence among Chinese Singaporeans. Previous studies have found differences in health status as well as variations in the occurrence of mood disorders according to ethnicity (Johnson-Lawrence et al., 2013). Further investigation of ethnic differences in mood is warranted among the Singaporean population, perhaps by mapping the incidence of negative mood profiles in our dataset to the incidence of mental health disorders at the national level. Such investigations would further inform policy makers and practitioners on potential preventive or protective measures as well as interventions for the vulnerable groups.

There were few significant variations in the distribution of mood profiles related to level of education, although the relatively small number of participants in the primary/secondary and postgraduate education groups should be noted. Previous literature suggests that level of education and socioeconomic status may have an influence on the mental health of individuals (Hudson, 2005; Eid et al., 2013) and therefore the relationship between education level and mood profiles might provide the focus for future investigations in a Singaporean context.

A significant finding from our study relates to the distribution of mood profile clusters in relation to participation in sport. Our findings showed a clear association between sport participation and mood responses. Those engaged in sport were over-represented in the positive iceberg mood profile and under-represented in the more negative inverse Everest, inverse iceberg, and submerged profiles. This association between sport participation and positive mood profiles might be related to the positive effects of sport on mood or the effects of mood on physical functioning (Morgan, 1985; Beedie et al., 2000; Terry and Lane, 2000), although the direction of effects cannot be ascertained in our study due to its correlational design. The study also found that a lack of participation in sport was associated with experiencing negative moods, as evident in the over-representation of the inverse Everest (the most negative profile, characterized by high scores for tension and fatigue, combined with very high scores for depression, anger, and confusion, and perhaps indicative of psychopathology), inverse iceberg, and submerged profiles.

Research has increasingly shown physical activity to be an effective intervention to promote physical and psychological benefits for both healthy and clinical populations (Penedo and Dahn, 2005; Thøgersen-Ntoumani et al., 2005; Biddle and Asare, 2011) and our results are certainly consistent with that burgeoning evidence base. Practitioners can be confident in the potential for sport and other forms of physical activity to be effective interventions to enhance mood and health, and the findings from our study can be used to inform health programs targeted at the different demographic groups. Given the inherent benefits of physical activity on mood and well-being, future studies could adopt experimental designs to evaluate the effectiveness of specific interventions (e.g., music listening; Terry et al., 2020) used to encourage and maintain participation in physical activity, with mood profiling used to evaluate the efficacy of interventions for promoting positive moods.

Despite our sample size of 1,444 participants, when participants were distributed across the six mood profiles the number of participants in some cells was very small (see Table 8). We acknowledge this as a limitation of our study and hence some findings, particularly those related to ethnicity and education level, should be treated with caution. Some of the less frequently reported profiles, notably the inverse Everest profile which was reported by fewer than 5% of participants, would require a much larger overall sample for detailed exploration. Larger and/or more targeted samples would allow researchers to investigate if certain profile types, especially those with a negative orientation, could predict physical or mental health deficits that might signal the need for detailed follow-up or intervention. The lack of planned follow-up with respondents reporting extremely negative mood profiles was another limitation, given that only email addresses were recorded, which participants could decline to provide. Future studies should consider carefully the kind of contact information to be collected, inform participants of potential follow-ups, and provide them a brief mood report as well as avenues for assistance if required.

In summary, when the previous validation of the BRUMS for use in a Singaporean context (Han et al., 2019) is considered in tandem with the replication of mood profiles in the present study, many potential applications of mood profiling are feasible. The mood profile clusters identified provide local practitioners with a way to better interpret BRUMS test scores. The utility and meaningfulness of mood profiles will increase further once associations are established with objective measures of performance, behavioral outcomes or mental health indicators. Such knowledge would inform practitioners how to use mood profiling more effectively in clinical and non-clinical settings. For example, practitioners could use mood profiling as a method to help individuals and teams to build a stronger understanding of whether certain mood profile types facilitate or debilitate performance, especially in occupations and work conditions that are demanding or high-risk, such as military environments, law enforcement, elite sport, aviation, and so on. There is also the possibility of using the BRUMS as a screening or monitoring tool for mental health purposes, once the therapeutic meaningfulness of each of the six mood profiles is established.

## Conclusion

The findings of the current study provide evidence that the mood profile clusters previously reported by Parsons-Smith et al. (2017) and Quartiroli et al. (2018) are also evident in the English-speaking Singaporean resident population, confirming the cross-cultural replicability of the clusters. The current study raises many research questions for future investigation that relate to the antecedents, correlates, and consequences of the six mood profile clusters, and how mood profiling can be used to, for example, predict performance outcomes or monitor mental health status. Results from this study set the stage for further research in Singapore to explore the practical, clinical, and theoretical implications of mood profiling.

## Data Availability Statement

The datasets generated for this study are available on request from the corresponding author.

## Ethics Statement

This study involving human participants was reviewed and approved by the Human Research Ethics Committee at the University of Southern Queensland (approval number: H17REA143). The participants provided informed consent to participate in this study.

## Author Contributions

All authors listed have made a substantial, direct and intellectual contribution to the work, and approved it for publication.

## Conflict of Interest

The authors declare that the research was conducted in the absence of any commercial or financial relationships that could be construed as a potential conflict of interest.
